# Worldwide cancer statistics of adults over 75 years old in 2019: a systematic analysis of the global burden of disease study 2019

**DOI:** 10.1186/s12889-022-14412-1

**Published:** 2022-10-28

**Authors:** Danhong Xiang, Shiwei Hu, Tianxiang Mai, Xinlu Zhang, Lan Zhang, Shengjie Wang, Keyi Jin, Jian Huang

**Affiliations:** 1 Department of Haematology, College of Medicine, The Fourth Affiliated Hospital, Zhejiang University, 322000 Zhejiang, People’s Republic of China; 2 Wenzhou Medical University, 325035 Zhejiang, People’s Republic of China; 3 Department of Haematology, College of Medicine, The First Affiliated Hospital, Zhejiang University, 310003 Zhejiang, People’s Republic of China; 4 Zhejiang Provincial Clinical Research Centre for Haematological Disorders, Hangzhou, 310003 Zhejiang People’s Republic of China

**Keywords:** Cancer burden, Adults over 75 years old, Incidence, Death rate, Trend

## Abstract

**Background and purpose:**

Cancer has become one of the major killers of humanity due to the number of people over the age of 75 increasing with population ageing. The aim of this study was to analyse the incidence and mortality rates in people over 75 of 29 cancer types in 204 countries and regions, as well as the trends from 1990 to 2019.

**Methods:**

Twenty-nine cancer types were collected from the Global Burden of Disease (GBD) 2019 database(https://vizhub.healthdata.org/gbd-results/). We collected global cancer data for 2019 in terms of sex, age, sociodemographic index (SDI), region, etc. The estimated annual percentage change (EAPC) was calculated to assess the trend of the cancer incidence and mortality rate from 1990 to 2019.

**Results:**

In 2019, the number of new cancer cases and deaths among people 75 and older was almost 3 and 4.5 times that of 1990, respectively. From 1990 to 2019, there was a slow rise in incidence and a slight decline in mortality. There were significant differences in the cancer burden based on sex, age, region, and SDI. The cancer burden in men was higher than in women. In addition, the cancer burden varied from region to region. The highest cancer burden occurred in high-income North America. In addition, the higher the SDI was, the greater the burden of cancer. The incidence of cancer in high SDI was approximately seven times that of low SDI, and the trend of increase in high SDI was obvious. However, the trend of mortality in high SDI was decreasing, while it was increasing in low SDI.

**Conclusions:**

The present study focused on the cancer burden in adults over 75 years old. The findings in the study could serve as the basis for an analysis of the types of cancers that are most prevalent in different regions. This is beneficial for strategies of prevention and treatment according to the characteristics of different countries and regions to reduce the burden of cancer in older adults.

**Supplementary Information:**

The online version contains supplementary material available at 10.1186/s12889-022-14412-1.

## Keypoints

- The incidence and death rate trends vary by cancer in adults over 75 years old.
- The cancer burden in adults over 75 years old was disproportionally greater among men than among women.
- The distribution and proportion of cancer in adults over 75 years old varied across geographic and SDI regions.
- The cancer burden in adults over 75 years old was much higher than the world average.

## Introduction

Noncommunicable diseases, including cancer, are the leading cause of death among older adults [1]. Cancer is more common in the older people than in any other age group. As the population is aging, it estimates that the number of people aged 65 and older will grow from 43 million in 2012 to 83 million in 2050, while the proportion of people aged 75 and older increased from 43.7% to 55%[2]. Accordingly, Negarten proposed classifying the elderly under the age of 75 years as the young-old and those who are 75 years or older as the old-old. Many studies on cancers used 75 years old as a cutoff [3, 4]. Adults aged 75 and older are generally referred to as older people. The increasing elderly population means that the cases and deaths from cancer will gradually increase worldwide. In addition, cancer in older people has its own unique characteristics, such as the distribution of cancer types, risk factors, cancer progression and treatment outcome. Moreover, due to the complexity and multidimensional nature of older cancer patients [5], they are significantly more vulnerable in physiology, mentality and economics than younger people and may require more care. To a large extent, these factors may affect the allocation of health care resources. Therefore, the profiles of the global burden of cancer among older people need to be depicted, which will be useful to guide health policies and improve cancer-related outcomes.

Some studies have found that the incidence of most cancers peaks at ages 75 to 95 [6–8]. However, research on cancers in older people has mainly been conducted in countries with a high sociodemographic index (SDI), especially the United States and several European countries [9, 10]. There has been no research focused on the global burden of cancer in individuals aged 75 and older who we called “older people”. In this study, we collected epidemiology of cancer data among those older people from the Global Burden of Disease (GBD) 2019 study from 1990 to 2019, including the incidence and mortality by sex, country, and region. Moreover, we calculated the estimated annual percentage change (EAPC) to analyse the trends of the incidence and mortality rate. An accurate evaluation of the worldwide burden and trends of cancer among the older people may have a positive impact on health policies.

## Data source and collection

We collected detailed information on cancers between 1990 and 2019 from the GBD (https://vizhub.healthdata.org/gbd-results/). The GBD 2019 database contains statistical data of 354 diseases in 204 countries and territories from 1990 to 2019 [11], among which we obtained the incidence and mortality rates of 29 cancers in people over 75 years of age. The 29 types of cancer in GBD include breast cancer, bladder cancer, colon and rectal cancer, leukaemia, lung cancer (including tracheal, bronchus, and lung cancer), etc. Then, they were analysed by gender, country, region, and sociodemographic index (SDI). At the same time, to compare the cancer incidence cases and cancer-related deaths with other age groups, we collected data for ages 0 to 14 years, 15 to 59 years, and 60 to 74 years. SDI is a measure of social development, and all countries are classified into five levels from 0 to 1: low SDI (0–0.454743), low medium SDI (0.454743–0.607679), medium SDI (0.607679–0.689504), medium high SDI (0.689504–0.805129) and high SDI (0.805129–1) (Supplementary Table S6). We analysed the incidence and mortality rates of all cancers and their trends on the background of the SDI and 21 geographic regions that were grouped by GBD 2019. All data was publicly available at https://vizhub.healthdata.org/gbd-results/.

## Statistical analysis

Reported estimates of prevalence, incidence, and mortality were entered in DisMod-MR 2.1 for analysis, which is a Bayesian meta-regression tool used in GBD 2019 to meta-analyse prevalence [12]. The EAPC is a summary and widely used measure of the rate trend over a specified interval. We used EAPC to assess the trends in age-standardized incidence and mortality. A regression line was fitted to the natural logarithm of the rates: y = α + βx + ε, where y = ln (rate), x = calendar year, and ε is the error term. EAPC = 100 × (exp(β) − 1), and its 95% confidence interval (CI) can also be obtained from linear regression models [13]. The cancer incidence or mortality was deemed to be an increasing trend if the EAPC estimation and the lower boundary of its 95% CI were both greater than 0. A decreasing trend meant the EAPC estimation and the upper boundary of its 95% CI were both less than 0. Otherwise, the incidence or mortality was deemed to be stable over time. All calculations were performed using R software (version 4.1.0) with ggplot2, maps, stringr, and readxl packages. The statistical significance was set at P < 0.05.

## Results

### Overview of the burden of cancer

The GBD 2019 data show that in 2019, the total number of cancer cases in the world over 75 years old was 6,746,260. The male incidence was 3302.68, while the female incidence was 1821.66 per 100,000 people, with a male: female ratio of 1.81. Cancer caused 3,487,482 deaths, with a male-to-female mortality of 1.72. A male predominance is a peculiarity of the cancer burden in aged 75 years and older. From 1990 to 2019, the incidence of cancer showed an upwards trend, whereas the mortality related to cancer showed a downwards trend. Among the 29 types of cancers, nonmelanoma skin cancer was the most common in men, accounting for 33.51%, followed by prostate cancer, lung cancer and colon and rectal cancer, accounting for 13.21%, 11.45% and 9.31%, respectively. Nonmelanoma skin cancer was also the most common in women at 33.71%, followed by colon and rectal cancer accounting for 11.74%, breast cancer accounted for 10.62% and lung cancer accounted for 8.46% (Tables 1 and 2).

**Table 1:** Worldwide incidence and rates by sex for the 29 cancer types in 2019 in adults over 75 years old and the change in the trends from 1990 to 2019

	**Both sexes**		**Male**		**Female**		**1990–2019** **EAPC** **No. (95% CI)**
	**Case**	**Rate per 100,000**	**Case**	**Rate per 100,000**	**Case**	**Rate per 100,000**	
Total cancer	6,746,260	2439.10	3,808,334	3302.68	2,937,926	1821.66	0.64(0.73–0.56)
Non-melanoma skin cancer	2,342,592	846.96	1,352,247	1172.70	990,344	614.06	1.36(1.52–1.21)
Colon and rectum cancer	699,550	252.92	354,675	307.58	344,876	213.84	0.39(0.66–0.13)
Lung cancer	684,689	247.55	436,009	378.12	248,680	154.19	0.90(1.17–0.62)
Prostate cancer	503,226	181.94	503,226	436.41	–	–	-0.07(0.24–0.37)
Stomach cancer	382,531	138.30	229,360	198.91	153,171	94.97	-0.65(-0.32–0.98)
Breast cancer	316,955	114.59	4988	4.33	311,968	193.44	-0.17(0.21–0.55)
Bladder cancer	199,743	72.22	147,699	128.09	52,044	32.27	0.26(0.75–0.24)
Pancreatic cancer	189,062	68.36	82,709	71.73	106,353	65.94	1.08(1.62–0.54)
Non-Hodgkin lymphoma	148,100	53.55	74,830	64.89	73,269	45.43	0.94(1.54–0.34)
Esophageal cancer	145,722	52.69	94,080	81.59	51,642	32.02	-0.11(0.44–0.66)
Liver cancer	132,488	47.90	78,330	67.93	54,158	33.58	-0.06(0.54–0.65)
Leukemia	130,715	47.26	68,928	59.78	61,786	38.31	0.56(1.18–0.06)
Kidney cancer	84,392	30.51	49,985	43.35	34,407	21.33	1.20(2.00–0.40)
Gallbladder and biliary tract	80,579	29.13	32,630	28.30	47,949	29.73	-0.26(0.49–1.00)
Lip and oral cavity cancer	69,899	25.27	38,349	33.26	31,550	19.56	0.33(1.19–0.51)
Malignant skin melanoma	68,290	24.69	36,981	32.07	31,309	19.41	1.72(2.64–0.81)
Uterine cancer	66,233	23.95	–	–	66,233	41.07	-0.04(0.78–0.86)
Multiple myeloma	52,776	19.08	26,692	23.15	26,084	16.17	0.44(1.41–0.52)
Brain and central nervous	50,752	18.35	23,800	20.64	26,953	16.71	1.57(2.64–0.51)
Ovarian cancer	49,447	17.88	–	–	49,447	30.66	-0.37(0.58–1.31)
Cervical cancer	45,564	16.47	–	–	45,564	28.25	-0.88(0.07–1.83)
Larynx cancer	36,108	13.05	30,017	26.03	6091	3.78	-0.23(0.91–1.35)
Thyroid cancer	24,888	9.00	8917	7.73	15,971	9.90	0.71(2.16–0.71)
Other pharynx cancer	22,414	8.10	15,921	13.81	6493	4.03	1.39(3.04–0.22)
Nasopharynx cancer	14,795	5.35	9731	8.44	5064	3.14	0.75(2.72–1.19)
Hodgkin lymphoma	13,056	4.72	7188	6.23	5868	3.64	0.44(2.44–1.52)
Mesothelioma	11,864	4.29	9087	7.88	2777	1.72	0.31(2.34–1.69)
Testicular cancer	2789	1.01	2789	2.42	–	–	1.11(5.80–3.37)
Other malignant neoplasms	177,040	64.01	89,167	77.33	87,873	54.49	1.10(1.66–0.55)

*EAPC* estimated annual percentage change

**Table 2:** Worldwide deaths and rates by sex for the 29 cancer types in 2019 in adults over 75 years old and the change in the trends from 1990 to 2019

	**both**		**Male**		**Female**		**1990–2019** **EAPC** **No. (95% CI)**
	**Case**	**Rate per 100,000**	**Case**	**Rate per 100,000**	**Case**	**Rate per 100,000**	
Total cancer	3,487,482	1260.90	1,923,318	1667.95	1,564,164	969.86	-0.05 (0.07 ~ -0.16)
Lung cancer	701,015	253.45	445,168	386.06	255,847	158.64	0.68 (0.95 ~ 0.42)
Colon and rectum cancer	468,185	169.27	226,604	196.52	241,581	149.79	0.01 (0.33 ~ -0.31)
Stomach cancer	346,650	125.33	199,465	172.98	147,185	91.26	-1.1 (-0.82 ~ -1.48)
Prostate cancer	308,160	111.41	308,160	267.24	–	–	-0.32 (0.07 ~ -0.70)
Pancreatic cancer	205,547	74.32	89,873	77.94	115,675	71.72	0.10 (1.52 ~ 0.48)
Breast cancer	182,595	66.02	3410	2.96	179,186	111.10	-0.48 (0.01 ~ -0.97)
Esophageal cancer	156,277	56.50	101,739	88.23	54,538	33.82	-0.45 (0.07 ~ -0.97)
Liver cancer	139,039	50.27	80,105	69.47	58,935	36.54	-0.54 (0.031 ~ -1.10)
Bladder cancer	125,997	45.55	89,449	77.57	36,548	22.66	-0.27 (0.34 ~ -0.87)
Leukemia	98,229	35.51	52,845	45.83	45,384	28.14	-0.05 (0.63 ~ -0.74)
Non-Hodgkin lymphoma	88,570	32.02	44,157	38.29	44,413	27.54	0.31 (1.06 ~ -0.43)
Gallbladder and biliary	74,599	26.97	29,795	25.84	44,804	27.78	-0.36 (0.42 ~ -1.13)
Kidney cancer	61,620	22.28	36,019	31.24	25,601	15.87	1.05 (1.98 ~ 0.12)
Ovarian cancer	52,315	18.91	–	–	52,315	32.44	-0.31 (0.61 ~ -1.23)
Lip and oral cavity cancer	49,820	18.01	26,858	23.29	22,962	14.24	0.37 (1.39 ~ -0.64)
Cervical cancer	48,158	17.41	–	–	48,158	29.86	-0.91 (0.02 ~ -1.82)
Multiple myeloma	45,876	16.59	22,733	19.71	23,144	14.35	0.23 (1.26 ~ -0.79)
Brain and central nervous	44,701	16.16	21,684	18.81	23,017	14.27	1.09 (2.18 ~ 0.00)
Non-melanoma skin cancer	30,240	10.93	16,243	14.09	13,997	8.68	0.92 (2.27 ~ -0.41)
Uterine cancer	29,990	10.84	–	–	29,990	18.60	-0.75 (0.42 ~ -1.91)
Larynx cancer	28,527	10.31	23,509	20.39	5018	3.11	-0.74 (0.49 ~ -1.96)
Malignant skin melanoma	22,378	8.09	11,604	10.06	10,774	6.68	0.46 (1.95 ~ -1.01)
Other pharynx cancer	20,239	7.32	14,369	12.46	5870	3.64	1.02 (2.71 ~ -0.64)
Thyroid cancer	16,561	5.99	6329	5.49	10,232	6.34	0.22 (1.94 ~ -1.47)
Mesothelioma	11,680	4.22	8791	7.62	2889	1.79	0.41 (2.48 ~ -1.62)
Nasopharynx cancer	10,145	3.67	6442	5.59	3703	2.30	-0.88 (1.19 ~ -2.91)
Hodgkin lymphoma	4364	1.58	2288	1.98	2076	1.29	-1.95 (0.96 ~ -4.78)
Testicular cancer	1254	0.45	1254	1.09	–	–	0.07 (6.70 ~ -6.14)
Other malignant neoplasms	114,750	41.49	54,423	47.20	60,326	37.41	0.13 (0.78 ~ -0.51)

*EAPC* estimated annual percentage change

**Fig. 1:**
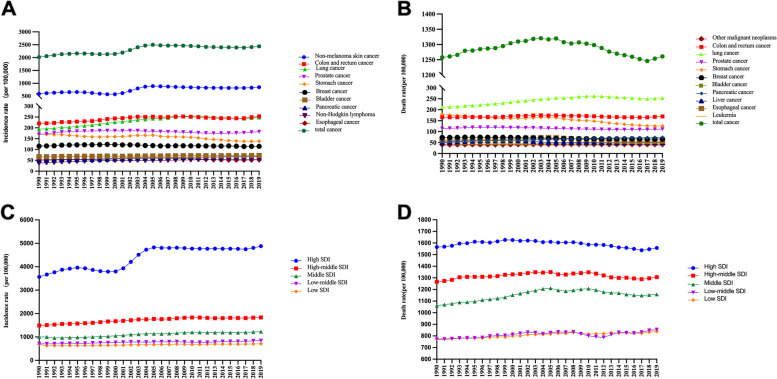
Worldwide trends of incidence (A, C) and death rate (B, D) by cancer type and SDI quintile in adults over 75 years old from 1990 to 2019. **a** Top 10 cancer types in terms of incidence; **b** Top 10 cancer types in terms of death rates; **c** Incidence rates in different SDI quintiles; **d.** Death rates in different SDI quintiles; SDI, socio-demographic index.

Among the other cancers, the incidence of nonmelanoma skin cancer (846.96/100,000), colon and rectal cancer (252.96/100,000), lung cancer (247.55/100,000), prostate cancer (181.96/100,000) and stomach cancer (138.30/100,000) were in the top five in 2019 (Fig. 1-A and Table 1). From 1990 to 2019, brain and central nervous system cancer (EAPC: 1.57), malignant melanoma (EAPC: 1.72), other pharynx cancer (EAPC: 1.39), nonmelanoma skin cancer (EAPC: 1.36), and kidney cancer (EAPC: 1.20) showed the most significant increases **(**Table 1). Lung cancer (253.45/100,000), colon and rectal cancer (169.27/100,000), stomach cancer (125.33/100,000), prostate cancer (111.41/100,000) and pancreatic cancer (74.32/100,000) had higher mortality in 2019 (Fig. 1-B and Table 2). Kidney cancer (EAPC: 1.05), brain and central nervous system cancer (EAPC: 1.09) and other pharynx cancer (EAPC: 1.02) had the steepest upward trend. The slight decrease in the mortality of cancer was due to the decrease in the mortality from stomach cancer (EAPC: -1.1), cervical cancer (EAPC: -0.91) and Hodgkin lymphoma (EAPC: -1.95) (Table 2).

## Heterogeneity of cancer types

**Fig. 2:**
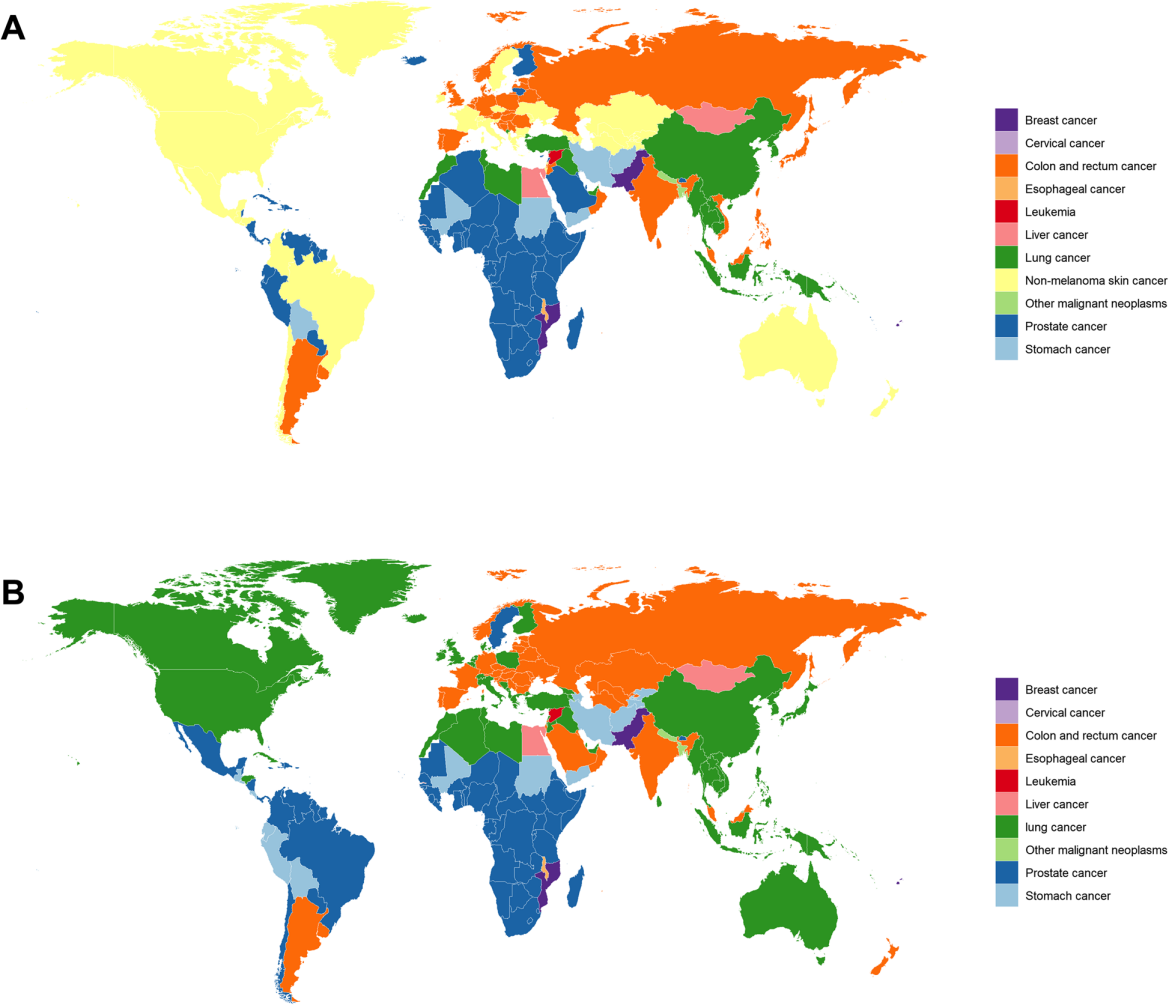
Global map of the most common cancer type by country in terms of incidence cases (**a**) and cancer-related death cases (**b**) in adults over 75 years old in 2019

We calculated the highest incidences of cancer in 204 countries and regions, and the most common cancers were breast cancer, cervical cancer, colon and rectal cancer, oesophageal cancer, leukaemia, liver cancer, lung cancer, nonmalignant skin cancer, other malignant neoplasms, prostate cancer, and gastric cancer (Fig. 2). Among these, the top five types of cancer, including prostate cancer, colon and rectal cancer, nonmalignant skin cancer, lung cancer and stomach cancer, had the highest incidence in 86 countries, 40 countries, 34 countries, 26 countries and 7 countries, respectively (Supplement S2-A). The highest mortality in 204 countries were from breast cancer, cervical cancer, colon and rectal cancer, oesophageal cancer, leukaemia, liver cancer, lung cancer, other malignant neoplasms, prostate cancer, and stomach cancer (Fig. 2). Prostate cancer was the most common cancer in 72 countries, and lung cancer had the highest mortality rate in 68 countries, followed by colon and rectal cancer. Gastric cancer was the highest in 37 countries and 16 countries, respectively (supplemental S2-B).

## Age-specific burden

**Fig. 3:**
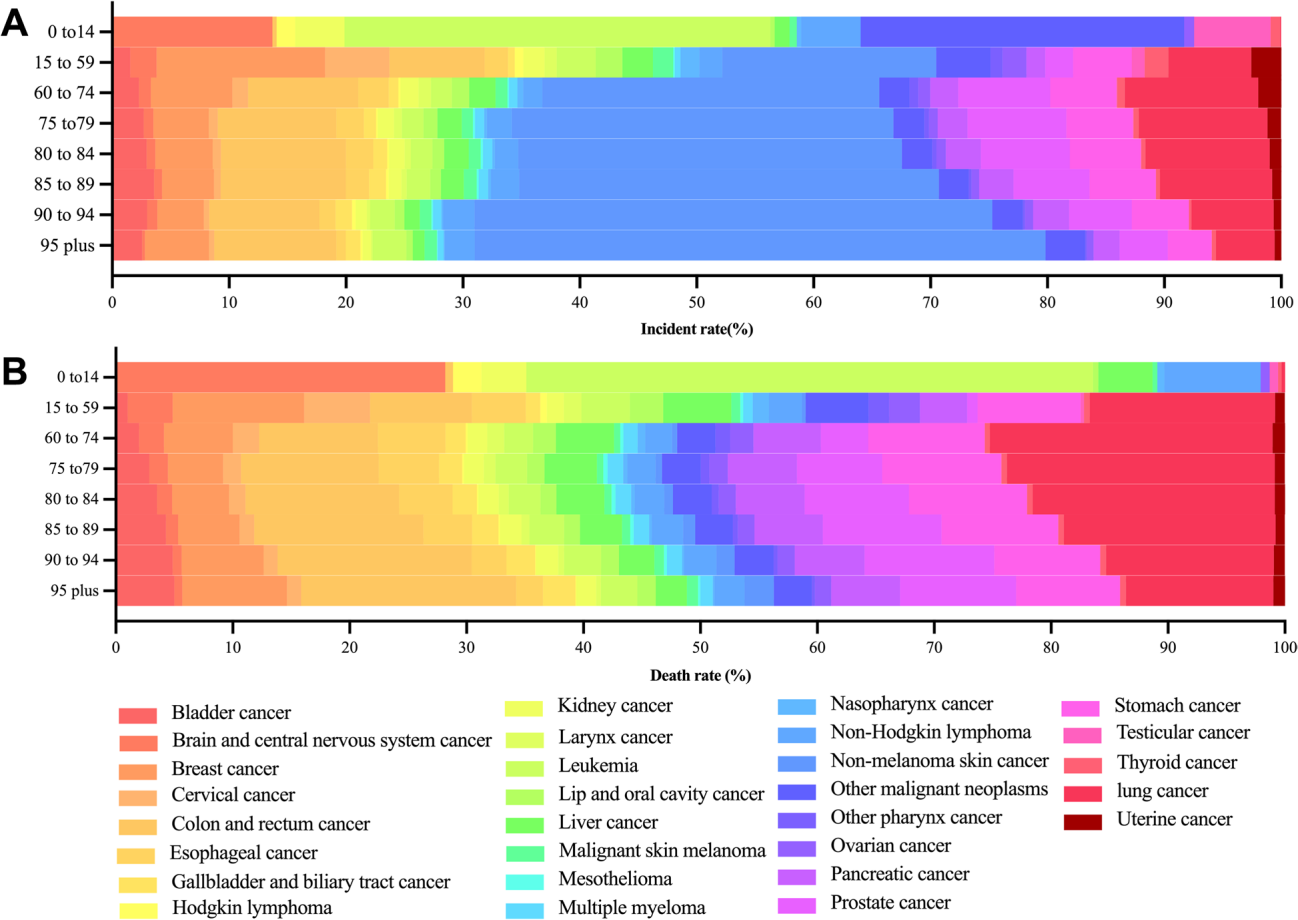
Worldwide distribution of cancer type by age group (**a**), incidence, and (**a**) death cases in 2019

According to the data, there were significant differences in the cancer incidence cases and cancer-related deaths among different age groups, and the burden of cancer in the older people was different from that in other age groups. In 2019, the number of cancer individual in those aged 75 and older accounted for 28.62% of the new cases, which was far higher than the 1.34% of the 0- to 14-year-old group and slightly lower than the 30.43% of the 25- to 59-year-old group and the 39.71% of the 60- to 75-year-old group. Individuals aged 75 and older account for a large proportion of the cancer burden. In the 0- to 14-year-old group, brain and central nervous system cancer, leukaemia, other malignant neoplasms, testicular cancer, and non-Hodgkin lymphoma were common, while breast cancer, nonmelanoma skin cancer, colon cancer and rectal cancer were common in the 15–59 age group. The most common type of newly diagnosed cancer in older people was nonmalignant skin cancer. In addition, we found that the number of nonmalignant skin cancers increased with age. Colon and rectal cancer, prostate cancer, and lung cancer also had high proportions (Fig. 3 and Supplement S3-A).

Approximately 34,877,482.176 older people died of cancer in 2019, accounting for 34.89% of all cancer-related deaths. Although nonmalignant skin cancer had the highest incidence, lung cancer was the top cause of death, followed by colon and rectal cancer and stomach cancer. The number of deaths caused by colon and rectal cancer, bladder cancer and prostate cancer increased with age. However, leukaemia killed nearly half of the children aged 0 to 14 who died from cancer, and more than a quarter of children died from CNS cancer. The leading causes of cancer death in youth and middle age were breast and lung cancer (Fig. 3 and supplement S3-A).

## Variations according to SDI levels

The global burden of cancer in older people varied by SDI region, being higher in high SDI regions than in low SDI regions. Notably, both the incidence and mortality increased with increasing SDI. For high SDI regions, the incidence and mortality of cancer were 4879.06 and 1557.61 per 100,000 people per year, respectively, followed by the low SDI regions with an incidence and mortality of 707.80 and 838.19 per 100,000 people per year, respectively. Briefly, the incidence and mortality of cancer in the high SDI regions was approximately 7.0 and 2.0 times greater, respectively, than that in the low SDI regions. With respect to the EAPC, the incidence had an upwards trend in all regions, especially in high-SDI regions, with a significant increasing trend of 1.11 (95% CI, 1.18 ~ 1.05), while the overall case fatality had a slight drop in high-SDI regions, contrary to other regions (Fig. 1-C and 1-D, Tables 3 and 4, Supplementary Table S4).

**Table 3:** Worldwide incidence and rates by SDI and regions for the 29 cancer types in 1990 and 2019 in adults over 75 years old and the change in the trends from 1990 to 2019

	1990		2019		1990–2019 EAPC No. (95% CI)
	Incidence case	Rate Per 100,000	Incidence case	Rate Per 100,000	
**Sex**					
Male	1,196,378	2675.84	3,808,334	3302.68	0.75(0.82–0.67)
Female	1,167,324	1614.05	2,937,926	1821.66	0.37(0.47–0.27)
**Socio-demographic index**					
High SDI	1,502,694	3564.95	4,054,431	4879.06	1.11(1.18–1.05)
High-middle SDI	497,666	1489.78	1,321,779	1832.94	0.71(0.81–0.61)
Middle SDI	237,209	1002.61	869,284	1226.38	0.90(1.02–0.77)
Low-middle SDI	92,668	713.75	321,938	843.02	0.51(0.66–0.36)
Low SDI	32,412	679.75	86,092	707.80	0.39(0.55–0.23)
**Region**					
**Africa**					
Central Sub-Saharan Africa	3013	826.99	8526	811.14	-0.15(-0.01–0.30)
Eastern Sub-Saharan Africa	11,081	711.61	29,604	804.88	0.41(0.56–0.26)
Western Sub-Saharan Africa	15,064	724.72	42,000	947.27	1.18(1.32–1.03)
Southern Sub-Saharan Africa	8383	1138.75	19,819	1317.37	0.31(0.42–0.20)
North Africa and Middle East	30,839	783.82	116,933	1051.80	1.33(1.47–1.19)
**America**					
Andean Latin America	7586	1250.06	30,461	1592.90	0.93(1.03–0.82)
Caribbean	12,805	1451.73	32,474	1721.31	0.67(0.77–0.56)
Central Latin America	30,385	1278.14	111,427	1409.05	0.19(0.30–0.08)
High-income North America	861,876	5938.18	2,504,561	10,039.24	2.09(2.14–2.05)
Southern Latin America	27,506	1787.97	65,153	2009.12	0.30(0.40–0.20)
Tropical Latin America	38,592	1603.37	137,467	1720.26	0.33(0.43–0.24)
**Asia**					
Central Asia	13,152	917.06	18,940	1123.02	1.13(1.26–1.00)
East Asia	233,569	1161.09	1,054,014	1682.69	1.41(1.53–1.30)
High-income Asia Pacific	138,754	2051.03	530,610	2375.63	0.56(0.65–0.48)
South Asia	64,304	590.04	247,053	651.39	0.11(0.27–0.06)
Southeast Asia	42,909	724.64	150,662	953.43	0.88(1.03–0.74)
**Europe**					
Central Europe	77,398	1510.93	166,601	1921.53	0.94(1.04–0.84)
Eastern Europe	105,504	1075.85	160,626	1228.98	0.63(0.75–0.51)
Western Europe	599,742	2395.00	1,198,818	2814.76	0.48(0.56–0.40)
**Oceania**					
Australasia	40,857	4639.68	119,373	5781.98	0.68(0.73–0.62)
Other Oceania countries	386	762.65	1138	877.43	0.54(0.68–0.39)

*EAPC* estimated annual percentage change

**Table 4:** Worldwide deaths and rates by SDI and regions for the 29 cancer types in adults over 75 years old in 1990 and 2019 and the change in the trends from 1990 to 2019

	1990		2019		1990–2019 EAPC No. (95% CI)
	Death cases	Rate per100,000	Death cases	Rateper 100,000	
**Sex**					
Male	766,361	1714.06	1,923,318	1667.95	-0.12 (-0.03 ~ -0.22)
Female	705,571	975.59	1,564,164	969.86	-0.12 (0.01 ~ -0.25)
**Socio-demographic index**					
High SDI	659,861	1565.44	1,294,350	1557.61	-0.09 (0.01 ~ -0.20)
High-middle SDI	423,098	1266.55	942,526	1307.02	0.03 (0.15 ~ -0.08)
Middle SDI	251,009	1060.94	820,792	1157.97	0.30 (0.42 ~ 0.18)
Low-middle SDI	100,080	770.84	325,973	853.59	0.25 (0.39 ~ 0.10)
Low SDI	37,031	776.63	101,952	838.19	0.26 (0.41 ~ 0.12)
**Region**					
**Africa**					
Central Sub-Saharan Africa	3449	946.69	9616	914.84	-0.18 (-0.06 ~ -0.34)
Eastern Sub-Saharan Africa	12,619	810.39	33,501	910.84	0.41 (0.55 ~ 0.27)
Western Sub-Saharan Africa	17,411	837.59	47,729	1076.46	1.12 (1.26 ~ 0.99)
Southern Sub-Saharan Africa	8636	1173.23	19,355	1286.56	0.16 (0.27 ~ 0.04)
North Africa and Middle East	32,419	823.96	104,206	937.32	0.71 (0.86 ~ 0.57)
**America**					
Andean Latin America	7397	1218.87	24,905	1302.33	0.30 (0.41 ~ 0.18)
Caribbean	11,584	1313.35	25,884	1372.01	0.25 (0.36 ~ 0.13)
Central Latin America	25,549	1074.72	78,425	991.72	-0.47 (-0.34 ~ -0.60)
High-income North America	219,492	1512.26	383,080	1535.53	-0.00 (0.10 ~ -0.11)
Southern Latin America	25,210	1638.74	51,973	1602.70	-0.14 (-0.04 ~ -0.24)
Tropical Latin America	27,459	1140.84	88,040	1101.74	-0.06 (0.06 ~ -0.18)
**Asia**					
Central Asia	11,642	811.79	16,270	964.74	1.02 (1.16 ~ 0.88)
East Asia	261,789	1301.36	872,566	1393.02	0.30 (0.41 ~ 0.19)
High-income Asia Pacific	101,071	1494.02	333,292	1492.21	-0.08 (0.02 ~ -0.19)
South Asia	74,114	680.06	269,561	710.74	-0.11 (0.05 ~ -0.27)
Southeast Asia	48,636	821.37	157,407	996.11	0.58 (0.71 ~ 0.44)
**Europe**					
Central Europe	67,372	1315.20	124,217	1432.68	0.35 (0.46 ~ 0.24)
Eastern Europe	91,583	933.89	121,626	930.58	-0.05 (0.08 ~ -0.19)
Western Europe	410,425	1638.99	692,756	1626.55	-0.17 (-0.07 ~ -0.27)
**Oceania**					
Australasia	13,630	1547.82	31,786	1539.60	-0.07 (0.04 ~ -0.17)
Other Oceania countries	446	881.81	1286	991.74	0.50 (0.63 ~ 0.36)

EAPC, estimated annual percentage change

**Fig. 4:**
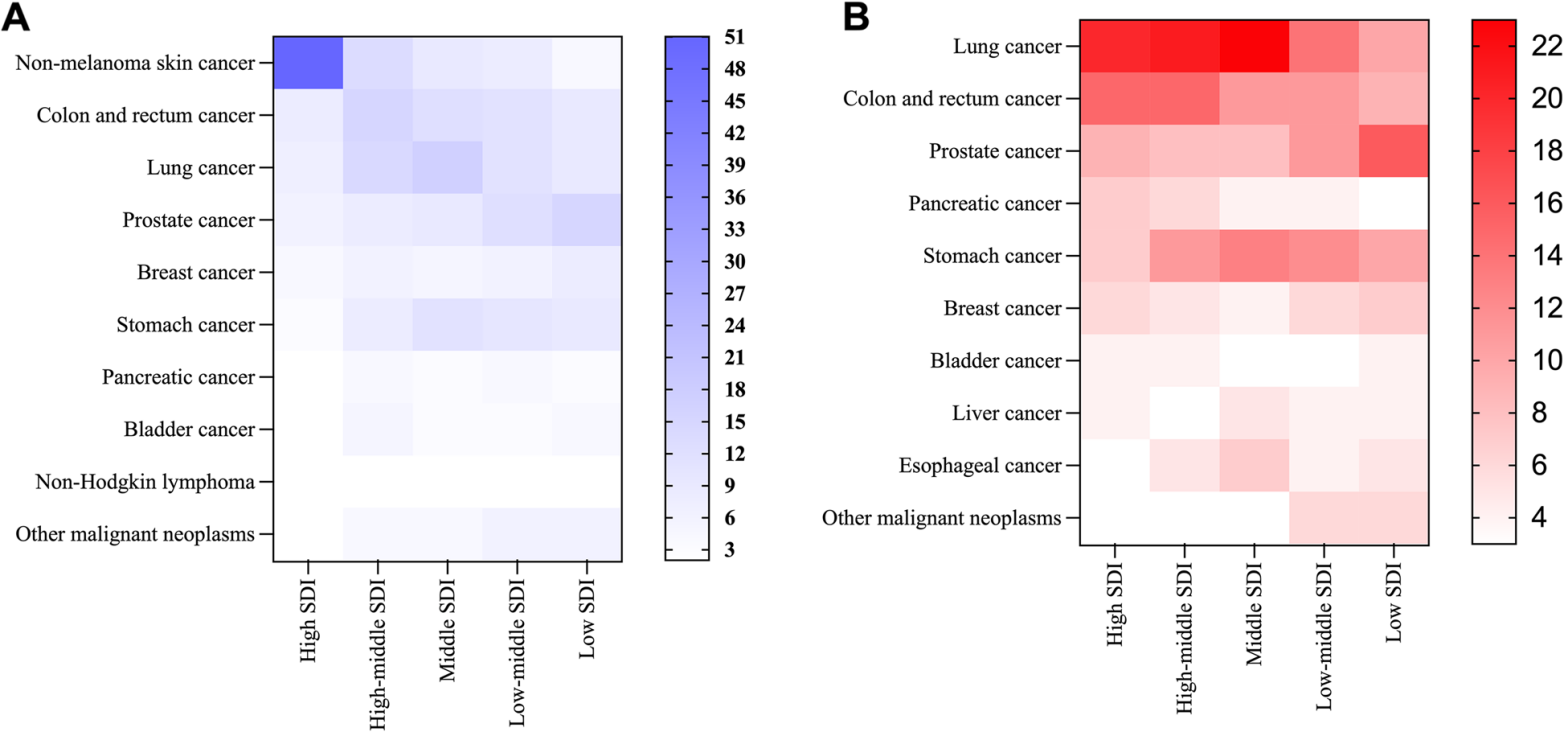
The proportion of top ten cancer type in different SDI quintiles in terms of incidence (**a**) and death (**b**) cases in adults over 75 years old in 2019

In addition to the analysis of total cancer based on SDI level, it was found that the top ten cancers in all the SDI regions followed certain patterns, among which the new cases and deaths accounted for three quarters or more of the estimated number (Fig. 4). Among these data, it was surprising that the top ten cancers were as high as 87.91% in high SDI regions, with nonmelanoma skin cancer alone accounting for 50.73% (rate 2475.11 per 100,000 people per year). Correspondingly, prostate cancer was the most frequently diagnosed cancer in the low SDI regions (15.98%, rate 119.77 per 100,000 people per year) and low-middle SDI regions (12.02%, rate 102.23 per 100,000 people per year). It was observed that the incidences of colon and rectal cancer, lung cancer, and prostate cancer were the top five across the SDI regions (Fig. 4). In terms of cancer profiles, lung cancer, colon and rectal cancer, prostate cancer, pancreatic cancer, and stomach cancer were among the most fatal cancers across all SDI levels. In high and high-middle SDI regions, lung cancer and colon and rectal cancer remained the largest contributors to the cause of cancer death, the estimated case fatalities of which were at least 3 times greater than those in the low SDI settings, while in the middle and low-middle SDI regions, lung cancer and stomach cancer ranked in the top two. For low SDI regions, prostate cancer was the leading cause of cancer-related death (16.26%, rate 136.26 per 100,000 people per year) (Fig. 4).

## Geographical differences in cancer burden

**Fig. 5:**
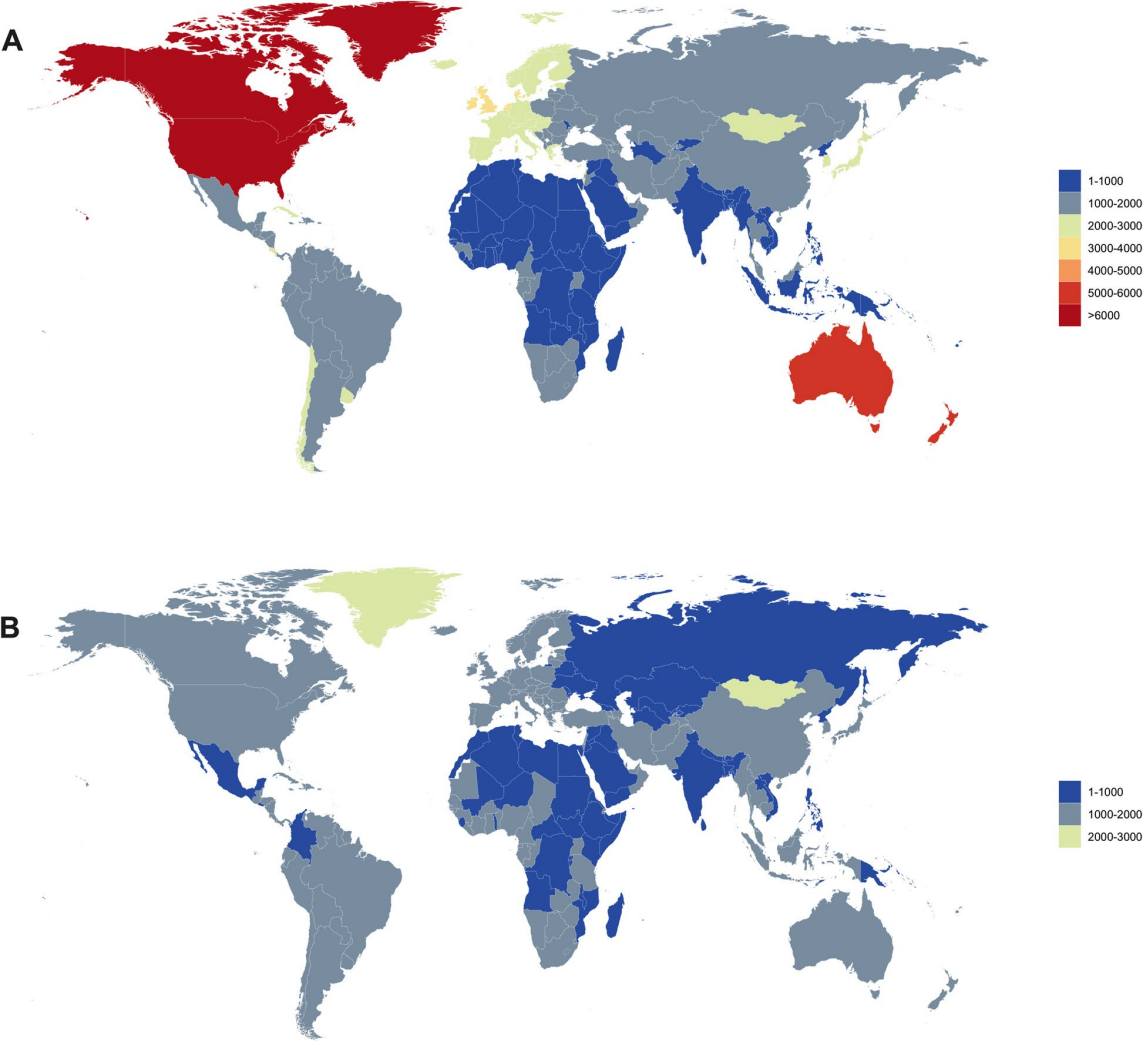
Global map of (**a**) incidence and (**b**) death rate for total cancers by country in adults over 75 years old in 2019

When assessed on a broader regional scale over the last three decades, the incidences of cancer in older people were greatest in most countries of North America, Southern Latin America, Western Europe, Australasia, and the high-income Asia Pacific, whereas Oceania, Africa, and part of Asia (especially South Asia and Southeast Asia) had the lowest incidences **(**Fig. 5). A significant upwards trend of incidences was observed in five regions, including high-income North America (2.09 per 100,000 people per year), East Asia (1.41 per 100,000 people per year), North Africa and the Middle East (1.33 per 100,000 people per year), western sub-Saharan Africa (1.18 per 100,000 people per year), and Central Asia (1.13 per 100,000 people per year). A slight downwards trend was observed only in central sub-Saharan Africa (Table 3, Supplementary Table S5A). Notably, regions with the highest incidences also ranked high in death rates, and the lowest three mortality burdens were in South Asia, Eastern Sub-Saharan Africa, and Central Sub-Saharan Africa (Fig. 5, Tables 3 and 4). Moreover, the mortality burden increased in almost half of the 21 GBD regions, especially in Western Sub-Saharan Africa and Central Asia, with EAPCs of 1.12 (95% CI, 1.26 ~ 0.99) and 1.02 (95% CI, 1.16 ~ 0.88), respectively (Table 4, Supplementary Table S5B).

**Fig. 6:**
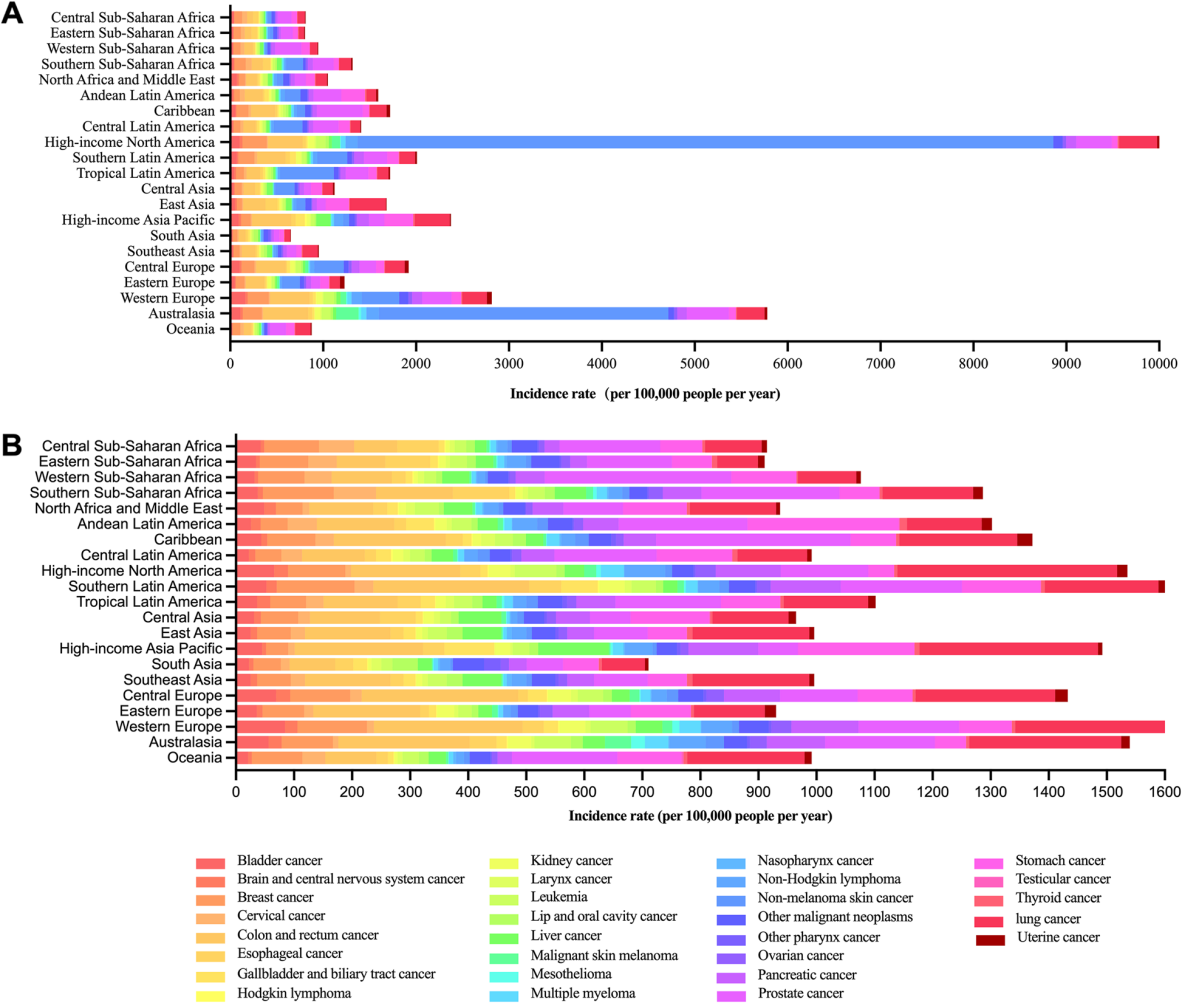
Incidence and death rates for the 29 cancer types by geographical region in adults over 75 years old in 2019. **a**. incidence rates; **b**. death rates

Additionally, the cancer profiles showed significant heterogeneity according to distinct regions. For instance, the incidence of nonmelanoma skin cancer varied by hundreds of times, with the lowest in Oceania (8.55 per 100,000 people per year) and the highest in high-income North America (7490.64 per 100,000 people per year) and Australasia (3117.33 per 100,000 people per year). The incidences of colon and rectal cancer, prostate cancer, and lung cancer varied approximately eightfold, ranging from 64.11 per 100,000 people per year in Central Sub-Saharan Africa to 484.25 per 100,000 people per year in Australasia, 58.61 per 100,000 people per year in South Asia to 491.93 per 100,000 people per year in the Caribbean, and 56.70 per 100,000 people per year in Eastern Sub-Saharan Africa to 410.44 per 100,000 people per year in high-income North America, respectively **(**Fig. 6A**)**. In the analysis of death rates, the greatest mortality burden of lung cancer, colon and rectal cancer, and pancreatic cancer was observed in almost the same regions, such as high-income Asia Pacific, Southern Latin America, high-income North America, and Central Europe, while the lowest mortality burden was concentrated in South Asia, Central Sub-Saharan Africa, and Eastern Sub-Saharan Africa. For instance, colon and rectal cancer had the highest mortality burden in Central Europe (269.64 per 100,000 people per year) and the lowest mortality burden in Central Sub-Saharan Africa (73.89 per 100,000 people per year). The mortality burden of lung cancer varied fivefold, ranging from 70.09 per 100,000 people per year in Eastern Sub-Saharan Africa to 378.47 per 100,000 people per year in high-income North America. However, it is worth noting that the mortality burden of prostate cancer had a higher death rate in the Caribbean and part of Africa (Western Sub-Saharan Africa and Southern Sub-Saharan Africa) and a lower death rate in South Asia and the high-income Asia Pacific, which was not consistent with several other cancers with high death rates (Fig. 6B).

## Discussion

To our knowledge, this is the first comprehensive analysis of cancer burden in adults over 75 years old across the world, since previous studies have focused on specific countries or all chronic diseases of ageing [9, 14, 15]. Our study filled the research gap for 29 cancers and reported their considerable variations of morbidity and mortality when stratified by age, sex, SDI, and geographical regions. We hope to provide recommendations for tailored cancer control strategies and mitigate the substantial pressure on health services.

Overall, almost 6.7 million new cancer cases were diagnosed in adults aged 75 older, and 3.5 million people died from cancer in 2019. A disproportionate part of this burden was borne by men, as their incidence and death rates were both higher than in older women. The incidences of total cancer have increased globally, and mortality has been decreasing since 2005. This phenomenon depends to a great extent on advances in diagnosis and treatment technology and the postponement of death with malignancies. The world's most common cancer was nonmelanoma skin cancer, and its incidence showed the most significant increasing trend [16]. Notably, nonmelanoma skin cancer was very common in North America, Australasia, and Central Asia. This could be due to the proximity to the ozone hole and exposure to high levels of ultraviolet light, which leads to a higher incidence of skin cancers [17, 18]. It was suggested to adopt inexpensive preventive strategies of known efficacy, such as avoiding sunlight and tanning and using more sunscreen. However, it was also observed that nonmelanoma skin cancer had a low mortality despite its high incidence. This is probably because basal cell carcinoma and squamous cell carcinoma rarely spread to other parts of the body and could be removed by simple surgery. On the other side, due to the growing proportion of the elderly population, its total number of death was higher than some other malignancies [16], which also need our attention.

There is a strong and positive relationship between the cancer incidence burden and higher SDI. One reason is that as a country transition socially, economically, and behaviourally with increasing human development, the cancer profiles also changed. The increase of industrialization-related cancers offset the decreased in infection-related cancers in more developed regions. For instance, the incidence and death rates and their trend of lung cancer and colon and rectal cancer both rank among the highest in the world and are closely related to changes in the environment and lifestyle. It has been acknowledged that high smoking rates and particulate matter (PM) 2.5 (PM ≤ 2.5 μm) are the leading contributors to the incidence of lung cancer [19–21], and the increasing incidence of colon and rectal cancer may be partially attributed to the prevalence of obesity, physical inactivity, and Western diet patterns [22]. Another reason for the high cancer incidence rate in high SDI corresponds with the availability and highly prevalent use of cancer screening. On the contrary, lacking cancer screening may course a lower cancer incidence and death rate in less developed regions. In addition, adolescents and young adults had higher incidence and lower mortality in high DSI, which may affect the incidence in older people for an apart of people who died when they were young [23]. Accessible to early detection programs and essential cancer medicines decreased the trend of death rate in high SDI, which represents that older people experience better survival prospects after diagnosis.

We know that older people with cancer were often accompanied by other chronic diseases, including cardiovascular diseases, respiratory diseases, and endocrine dysfunction, resulting in a worsened condition to tolerate the treatment, and leading to other accidents. So, it should be noted that the implication of cancer burden in older people are beyond the number of cancer cases and deaths. Therefore, it is particularly important to take targeted preventive measures, which have been proven to be effective at older ages as well. Studies have demonstrated that smoking cessation lowers the burden of lung cancer among older adults [24], and moderate physical activity reduces the burden of colon and rectal cancer at older ages [25]. Additionally, the higher the level of SDI, the greater the cancer burden among older adults. It was observed that high SDI regions had the greatest cancer burden, with 7 times the incidence and 2 times the death rate compared with low SDI regions, which maybe due to advanced detection techniques. High SDI regions had a higher incidence of nonmelanoma skin cancer, and low SDI regions had a higher incidence rate of prostate cancer. Colon and rectal cancer, lung cancer, and prostate cancer were common in all SDI. Thus, it is of great importance to assess the specific needs and implement optimal cancer control strategies for older people according to the cancer profiles in different regions.

It is worth pointing out that screening for cancer could reduce the burden of cancer effectively, cutting back on catastrophic health expenditures and long-term costs for a household [26]. However, cancer screening is often overlooked or discouraged in adults over 75 years old due to concerns about excessive anxiety, complications related to the screening test, excessive medical treatment and even false-negative results [27]. There are other impediments, such as knowledge of cancer prevention, healthy life expectancy, and state of the economy, all of which influence individual choices [28]. It should also be noted that the disease status of older people is more complex because there is substantial heterogeneity among them in terms of comorbidities, and the coexistence of diseases may influence the diagnosis and management of cancer. Therefore, departments of health should follow the recommendations of the International Society of Geriatric Oncology and conduct a comprehensive evaluation of older cancer patients, including mental health status, comorbidity cognition, functional status, and social status and support [29]. Additionally, the relevant department should make comprehensive considerations based on economy, technology, benefit, and other factors, encourage more randomized clinical trials taking this population into account and ensure rational allocation of existing or given resources to carry out more regular cancer screening of older people.

The quality and availability of the data sources determines the credibility of our research to a large extent. Although the GBD has the advantage of analysing data in areas where actual data are unavailable, in less developed countries where registration systems and verbal autopsies may be missing, estimates rely on predictive covariates or trends from adjacent areas, leading to analytical uncertainty. Moreover, evolution in diagnostic criteria, ascertainment bias of causes of death and underreporting can bring about underestimation or overestimation of cancer-related data. Finally, the GBD studies cannot be categorized by race, resulting in a lack of analysis using racial profiling.

## Conclusion

In summary, the global cancer burden among adults over 75 years old shows great heterogeneity in terms of sex, age, SDI, and GBD regions. In the last three decades, despite advances in the diagnosis and treatment of cancer, the cancer burden of the elderly remains considerable in high SDI regions. These results are partially due to the influence of different risk factors, social and economic conditions, lifestyles and health policies, providing a direction for policy-makers to adopt strategies based on regional characteristics and achieve the global targets of improving equity in cancer care.

## Supplementary Information

**Additional file 1.**

## Data Availability

The datasets and materials used and/or analysed during the current study are available from the Global Health Data Exchange query tool (http://ghdx. whealthdata.org/gbd-results-tool). All data was publicly available at https://vizhub.healthdata.org/gbd-results/.
