# Supplementary material for: Worldwide cancer statistics of adults over 75 years old in 2019: a systematic analysis of the global burden of disease study 2019
Source: BMC Public Health. 2022 Oct 28;22:1979. doi: 10.1186/s12889-022-14412-1 (PMC9617321; doi:10.1186/s12889-022-14412-1)
Supplement: Supplementary file 1 — Additional file 1. [file 12889_2022_14412_MOESM1_ESM.docx]

**Supplementary Table S1. Worldwide incidence (A) and deaths (B) of the top 10 cancer types in adults over 75 years old from 1990 to 2019.**

A

B

SDI, socio-demographic index.

**Supplementary Table S6. Included countries (n=204) according to the five-level socio-demographic index (SDI) in 2019**

**Low=0<SDI≤0.454743, Low-Middle= 0.454743<SDI≤0.607679, Middle=0.607679<SDI≤0.689504, High-Middle=0.689504<SDI≤0.805129, High=0.805129<SDI≤1)**

| **Low(n=33)** | **Low-Middle (n=42)** | **Middle (n=41)** | **High-Middle (n=47)** | **High (n=41)** |
| --- | --- | --- | --- | --- |
| **Pakistan** | **Mongolia** | **Thailand** | **Israel** | **Switzerland** |
| **Haiti** | **Belize** | **Panama** | **Poland** | **Norway** |
| **Rwanda** | **Kyrgyzstan** | **China** | **Italy** | **Monaco** |
| **Tanzania** | **Dominican Republic** | **Equatorial Guinea** | **Malta** | **Germany** |
| **Nepal** | **Tuvalu** | **Jamaica** | **Virgin Islands** | **Luxembourg** |
| **Togo** | **Palestine** | **Azerbaijan** | **The Bahamas** | **Andorra** |
| **Yemen** | **Federated States of Micronesia** | **Albania** | **Croatia** | **Denmark** |
| **Côte d'Ivoire** | **eSwatini** | **Costa Rica** | **Greece** | **San Marino** |
| **Solomon Islands** | **El Salvador** | **South Africa** | **Hungary** | **Netherlands** |
| **Uganda** | **Congo (Brazzaville)** | **Tunisia** | **Montenegro** | **United Arab Emirates** |
| **The Gambia** | **Bolivia** | **Iraq** | **Oman** | **South Korea** |
| **Eritrea** | **India** | **Turkmenistan** | **Northern Mariana Islands** | **Canada** |
| **Madagascar** | **Maldives** | **Saint Lucia** | **Serbia** | **Sweden** |
| **Papua New Guinea** | **North Korea** | **Iran** | **Spain** | **Japan** |
| **Senegal** | **Ghana** | **Grenada** | **Bulgaria** | **Iceland** |
| **Malawi** | **Morocco** | **Cuba** | **Cook Islands** | **Taiwan (province of China)** |
| **DR Congo** | **Marshall Islands** | **Fiji** | **Greenland** | **Ireland** |
| **Liberia** | **Tajikistan** | **Indonesia** | **Romania** | **Singapore** |
| **South Sudan** | **Kiribati** | **Egypt** | **Chile** | **USA** |
| **Guinea-Bissau** | **Guatemala** | **Gabon** | **Trinidad and Tobago** | **Finland** |
| **Benin** | **Cape Verde** | **Algeria** | **Bahrain** | **Belgium** |
| **Sierra Leone** | **Myanmar** | **Mexico** | **Turkey** | **Kuwait** |
| **Afghanistan** | **Nicaragua** | **Peru** | **Saint Kitts and Nevis** | **Austria** |
| **Ethiopia** | **Sudan** | **Samoa** | **Belarus** | **UK** |
| **Guinea** | **Nigeria** | **Ecuador** | **North Macedonia** | **Lithuania** |
| **Mozambique** | **Timor-Leste** | **Brazil** | **Portugal** | **Cyprus** |
| **Burundi** | **Kenya** | **Paraguay** | **Antigua and Barbuda** | **Slovenia** |
| **Central African Republic** | **Lesotho** | **Suriname** | **Barbados** | **New Zealand** |
| **Mali** | **Zambia** | **Tonga** | **Palau** | **Australia** |
| **Burkina Faso** | **São Tomé and PrÍncipe** | **Botswana** | **Malaysia** | **Estonia** |
| **Chad** | **Honduras** | **Colombia** | **Ukraine** | **France** |
| **Niger** | **Mauritania** | **Uzbekistan** | **Jordan** | **Qatar** |
| **Somalia** | **Laos** | **Saint Vincent and the Grenadines** | **Dominica** | **Czech Republic** |
|  | **Cameroon** | **Tokelau** | **Seychelles** | **Brunei** |
|  | **Vanuatu** | **Philippines** | **Kazakhstan** | **Latvia** |
|  | **Bangladesh** | **Syria** | **Bosnia and Herzegovina** | **Puerto Rico** |
|  | **Zimbabwe** | **Guyana** | **American Samoa** | **Bermuda** |
|  | **Angola** | **Nauru** | **Niue** | **Guam** |
|  | **Cambodia** | **Vietnam** | **Libya** | **Slovakia** |
|  | **Djibouti** | **Namibia** | **Argentina** | **Russia** |
|  | **Bhutan** | **Venezuela** | **Lebanon** | **Saudi Arabia** |
|  | **Comoros** |  | **Mauritius** |  |
|  |  |  | **Georgia** |  |
|  |  |  | **Uruguay** |  |
|  |  |  | **Moldova** |  |
|  |  |  | **Sri Lanka** |  |
|  |  |  | **Armenia** |  |
